# Supplementary material for: Externally Provided Rewards Increase Internal Preference, but Not as Much as Preferred Ones Without Extrinsic Rewards
Source: Comput Brain Behav. 2024 Feb 21;8(1):71–91. doi: 10.1007/s42113-024-00198-5 (PMC13298676; doi:10.1007/s42113-024-00198-5)
Supplement: Supplementary file 1 — Supplementary file1 (PDF 356 KB) [file 42113_2024_198_MOESM1_ESM.pdf]

Externally provided rewards increase internal preference, but not as much as preferred ones without extrinsic rewards

Jianhong Zhu[1*], Kentaro Katahira[2], Makoto Hirakawa[1], Takashi Nakao[1]

[1] Graduate School of Humanities and Social Sciences, Hiroshima University, 1-1-1 Kagamiyama, Higashi-hiroshima, Hiroshima, 739-8524, Japan

[2] Human Informatics and Interaction Research Institute, National Institute of Advanced Industrial Science and Technology (AIST) Tsukuba, 1-1-1 Higashi, Tsukuba, Ibaraki, 305-8566, Japan

* Corresponding author. E-mail: zhujianhong1995@gmail.com

Computational model analysis of the EDM task

We performed the computational model analysis of behavior data in the EDM task. In this study, because of the experimental design of the EDM task (feedback from the chosen option was independent of feedback from the rejected option), we chose to use a reinforcement learning (RL) model that only updated the value of the chosen options (RL 1; Equation s1). However, we cannot exclude participants from updating the value of rejected options at the same time. To confirm whether the EDM task behavior data in this study is more appropriate for the model used in this study (i.e., RL 1), we compared it with the model that updated both the value of chosen and rejected options (RL 2; Equation s2), which assumed that feedback from chosen options was associated with feedback from rejected options and had the opposite feedback.

The value of the chosen option was learned in the traditional reinforcement learning model through a set of previous behavioral outcomes and used to determine a later choice [1,2]. The learning process of the RL 1 and RL 2 models is written as follows:

Equation s1 $$V_i^{EDM}(t+1) = \begin{cases} V_i^{EDM}(t) + \alpha\left(r(t) - V_i^{EDM}(t)\right) & \text{if } i \text{ was chosen} \\ V_i^{EDM}(t) & \text{if } i \text{ was rejected} \end{cases}$$

Equation s2 $$V_i^{EDM}(t+1) = \begin{cases} V_i^{EDM}(t) + \alpha\left(r(t) - V_i^{EDM}(t)\right) & \text{if } i \text{ was chosen} \\ V_i^{EDM}(t) + \alpha\left((1 - r(t)) - V_i^{EDM}(t)\right) & \text{if } i \text{ was rejected} \end{cases}$$

For RL 2 model, the value V^{EDM} ($0 \leqq V^{EDM} \leqq 1$) of both the chosen item and the rejected item (*i*) was updated in each trial (*t*). As participants could not know whether the rejected item would be rewarded in each trial, the value of the rejected item was not updated in the RL 1 model, and the updated V^{EDM} was kept constant until the trial in which the stimulus is presented.

Since novel contour shapes were used in the behavioral experiment, the initial values of the stimuli were set to the same value [3,4,5]. The initial value (V^{EDM}) at the beginning of the experiment was set to 0. The degree of reward in the trial *t* was defined by r ($0 \leqq r \leqq 1$). $r(t) - V_i^{EDM}(t)$ and $(1 - r(t)) - V_i^{EDM}(t)$ represented prediction error in trial *t*. The learning rate α ($0 \leqq \alpha \leqq 1$) is a parameter that determines the degree of updating V^{EDM} in one trial. The calculation method of the probability of choice and the estimation method of the parameters in the RL models were the same as those in the CBL models in this study.

We fit these models with the same simulation and behavior data as this study. We used the same settings as the actual experimental design when generating the artificial behavioral dataset. In each model, we generated artificial data with 4 stimuli and 204 trials for 38 people. The range of parameters generated was the same as that of the CBL model used in this study.

Fig s1 shows the results of parameter recovery for each RL model. We confirmed strong consistency between the set parameter values (simulated) to

generate artificial behavioral data and the estimated values (fitted) by fitting the model generating the data in all RL models (rs > .751).

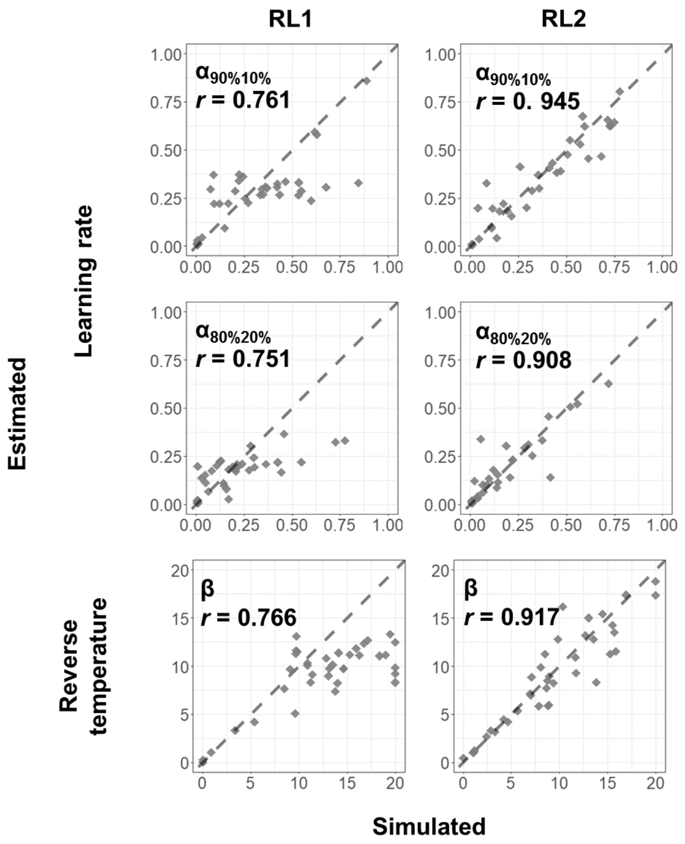

Fig s1. Results of parameter recovery simulation. This simulation was conducted to confirm whether each model could be well estimated as the set value of each parameter. The correlation coefficient between simulated and fitted was shown as parameter recovery indices.

Table s1 shows the results of model recovery. When RL 1 and 2 were the true models, there were very strong supports for each model compared to another model (BF_{12} = 1.725×10^{65}, BF_{21} = 3.154×10^{45}).

Table s1. Results of WBIC for model recovery

Simulated \ Fitted	RL 1	RL 2
RL 1	**2013.397**	2163.61
RL 2	2408.996	**2304.231**

Note: Bold numbers in the table represent the WBIC values of the models that best fit the artificial data.

After fitting the actual EDM behavioral data to these two RL models, the results presented in Table s2 indicated strong evidence to support RL 1 as compared to another model ($BF_{12} = 6.471 \times 10^{16}$). These results indicated that the RL model used in this study was consistent with the experimental design of the EDM task and behavioral, which only updated the value of the chosen option.

Table s2. WBIC results of EDM behavioral data fit with two types of RL models

Model	WBIC
**RL 1**	**2389.790**
RL 2	2428.498

Note: Bold numbers in the table represent the WBIC values of the model that best fit the behavioral data.

Finally, we examined the estimated value of each stimulus type at the end of the EDM trials (Fig s2) to confirm that value had been learned in EDM from the results of the RL 1 model analysis. Although we validated whether participants learned value from the EDM task through correct response rate analysis, final values estimated by the RL 1 model are also reported for reference. To confirm that high probability reward stimuli were more valuable than low probability reward stimuli at

the end of EDM, we compared those values. The results showed that the final value of any one high probability reward stimulus was higher than that of any one low probability reward stimulus (*t*s(37) < 24.602, Holm-adjusted *p*s < .001, *d*s > .609). Furthermore, the final value of stimuli rewarded with 90% probability was higher than that of 80% ($t(37) = 2.642$, Holm-adjusted $p = .012$, $d = .609$, 95% CI = 0.032, 0.243), and that of 10% was lower than that of 20% ($t(37) = -0.062$, Holm-adjusted $p = .001$, $d = -.467$, 95% CI = -0.096, -0.029).

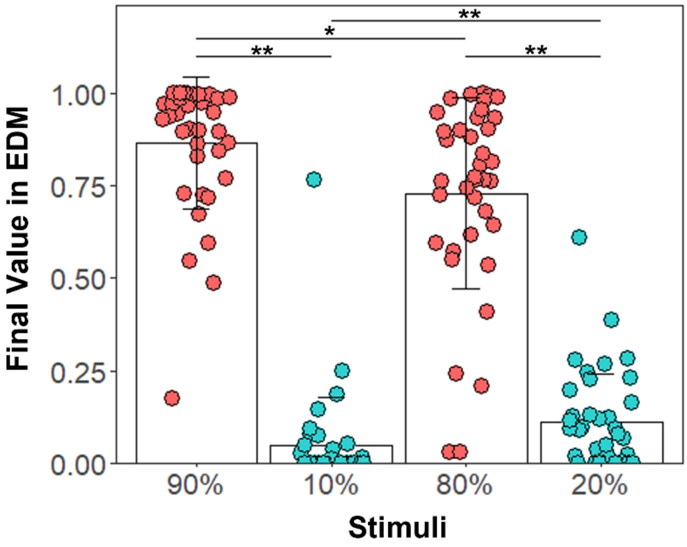

Fig s2. Mean estimated value of each stimulus type at the end of the EDM task.
* $p < .05$, ** $p < .001$.

One may argue that the final value of stimuli in the EDM estimated by the RL model is different from their initial value in the IDM estimated by the CBL model (see main manuscript Fig 3a, Fig s2), and the difference between the stimuli with high and low reward probabilities appears smaller in Fig 3a than in Fig s2. Notably, Fig 3a and Fig s2 show the results of estimating the data in different tasks with different

models and are the relative but not absolute values of the stimuli in each model or task. The values of the stimuli estimated by the different models were independent, and the value estimated by each stimulus was the relative value of the other stimuli within the same task. Therefore, a direct comparison of the estimates for different tasks using different models is not meaningful. Fig s2 confirms that high-probability stimuli are learned to be more valuable than low-probability stimuli. Meanwhile, Fig 3a shows that the initial value of HP (the stimulus that was the high-probability reward stimulus in the EDM) in the IDM was higher than that of LP (the stimulus that was the low-probability reward stimulus in the EDM). As these results reflect experimental manipulations, we can conclude that the difference in the value between HP and LP in EDM was reflected in the initial value of IDM, even though the estimated absolute values of these stimuli were different between RL and CBL.

Furthermore, one may propose that the final value of the EDM should be used as the initial value in the model analysis of the IDM. The contamination of errors in the estimated values is evident from the fact that in the parameter recovery simulation, there are errors between the parameters used to generate artificial data and the estimated parameters (Fig s1). To avoid the impact of errors on the IDM, we analyzed the IDM without including the estimates from the EDM.

References

1 Watkins, C. J., & Dayan, P. Q-learning. *Mach. Learn.* **8**, 279-292 (1992).

2 Sutton, R. S., & Barto, A. G. *Reinforcement learning: An introduction* (MIT Press, 2018).

3 Ohira, H. *et al*. Regulation of natural killer cell redistribution by prefrontal cortex during stochastic learning. *NeuroImage* **47**, 897–907 (2009).

4 Ohira, H. *et al*. Brain and autonomic association accompanying stochastic decision-making. *NeuroImage* **49**, 1024–1037 (2010).

5 Kunisato, Y. *et al*. Effects of depression on reward-based decision making and variability of action in probabilistic learning. *J. Behav. Ther. Exp. Psychiatry* **43**, 1088–1094 (2012).

The comparison between the RL and CBL models with the random choice model

For reference, we additionally included comparisons of the RL and CBL models used in the EDM and IDM with random choice models. In the random choice models, the probability of choice was 0.5 among all the options, and the value of the selected option was not updated through choice. By fitting the behavioral data to both random choice and actual models (i.e., RL 1 in EDM and Model 2 in IDM), we obtained a general goodness-of-fit for the models used in this study. In both the EDM and IDM, the results of fitting the actual behavioral data to all models indicated that the model used in this study had a better fit than the random choice models (Table s3; $BF_{EDM} = 5.209 \times 10^{1292}$, $BF_{IDM} = 4.130 \times 10^{256}$).

Table s3. The results of the WBIC for different models fitted to the behavior data of EDM and IDM, respectively.

Behavior data	Model	WBIC
EDM	**RL 1**	**2389.91**
EDM	Random choice	5373.28
IDM	**Model 2**	**2174.78**
IDM	Random choice	2765.66

Note: Bold numbers in the table represent the WBIC values of the model that best fit the behavioral data.
